# Penetrating hypopharyngeal foreign body impalement of the thyroid gland: A case report of rare complication of ingested fish bone

**DOI:** 10.1016/j.ijscr.2024.109851

**Published:** 2024-06-06

**Authors:** Marwa AlTarayra, Khalil N.M. Abuzaina, Ahmed M.I. Aljodi, Sulaiman Fakhouri, Ammar W.M. Hassouneh

**Affiliations:** a Governmental Hebron Hospital (GHH), Palestine; b General Surgery, Hebron University, Palestine; c Head of Surgical Oncology Unit, GHH, Palestine; d General Surgery, Palestine Polytechnique University, Palestine; e Radiologist, Governmental Hebron Hospital, Palestine

**Keywords:** hypopharyngeal perforation, foreign body ingestion, Fish bone, Case report

## Abstract

**Introduction and importance:**

Foreign body ingestion complicated by hypopharyngeal perforation is an uncommon but potentially life-threatening condition. Early recognition and appropriate management are crucial to prevent serious complications. We present an extremely rare case highlighting the importance of this clinical entity.

**Case presentation:**

A 60-year-old female presented with odynophagia 10 days after ingesting fish and chicken. Imaging revealed a linear foreign body penetrating through the left lateral hypopharyngeal wall into the left thyroid lobe, with surrounding inflammatory changes. The patient underwent neck exploration, which identified a sharp fishbone lodged in the postero-medial aspect of the left thyroid lobe, necessitating a left hemithyroidectomy for removal.

**Clinical discussion:**

To our knowledge, this is the first reported case of hypopharyngeal perforation by an ingested foreign body penetrating the thyroid gland itself. Despite its rarity, early recognition is crucial to prevent complications like abscess, mediastinitis, and mortality. A high index of suspicion is needed in patients with odynophagia or neck pain after ingesting fish. Advanced imaging and surgical intervention may be required for the management of larger perforations or those involving surrounding structures.

**Conclusion:**

This unique case highlights an extremely rare presentation of hypopharyngeal perforation with extension into the thyroid gland caused by an ingested fish bone. Prompt diagnosis through appropriate imaging and treatment with surgical exploration and foreign body removal was key to ensuring a positive outcome. Increased awareness of this potential complication is essential among clinicians.

## Highlights

• Extremely rare case of hypopharyngeal perforation by ingested fish bone penetrating into the left thyroid lobe
• First reported case of its kind
• Required neck exploration and left hemithyroidectomy for foreign body removal
• Early recognition is crucial to prevent complications like abscess/mediastinitis.
• Advanced imaging and surgical management needed for larger perforations/involvement of surrounding structures

## 1 Introduction

Foreign body ingestion is a common complaint frequently seen in the emergency department. Which, sharp objects may get lodged in the aerodigestive tract and subsequently cause a perforation. Although perforation of the hypopharynx is rare, early recognition and treatment is crucial to decrease the associated morbidity [1,2].

This case report presents an extremely rare case of hypopharyngeal perforation piercing the left thyroid lobe in a 60-year-old female 10 days after fish bone ingestion. A neck CT scan showed a foreign body penetrating the hypopharyngeal wall throughout the left thyroid lobe. She underwent neck exploration to remove the sharp object and subsequently a left hemithyroidectomy was performed.

## 2 Case presentation

A 60-year-old female presented to the ER complaining of odynophagia. These symptoms developed 10 days after ingestion of fish and chicken. There were no other related complaints (i.e., hematemesis, drooling, vomiting, choking, cyanosis, cough, dyspnea, or hemoptysis). The patient's medical and surgical histories were only significant for bronchial asthma.

On physical examination, she was hemodynamically stable and unremarkable except for mild tenderness over the left anterior neck above the level of the thyroid cartilage. A complete blood count and C-reactive protein level were obtained. The complete blood showed hemoglobin of 12.2 g/dL and a white blood cell count of 10.6 × 109/L. C-reactive protein was elevated at 9.4 mg/L.

**Fig. 1:**
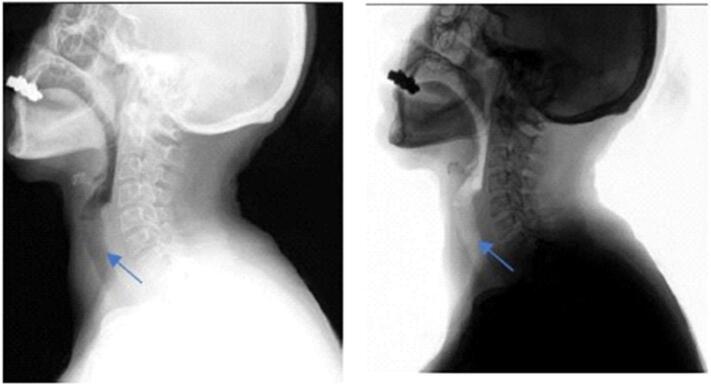
Lateral neck radiograph showed a linear radiopacity in the region of cricopharynx suggestive of a foreign body; Normal thickness of retropharyngeal and retrotracheal space.

The lateral neck radiograph showed a linear radiopacity in the region of the oropharynx suggestive of a foreign body; Normal thickness of retropharyngeal and retrotracheal space as shown in Fig. 1.

**Fig. 2:**
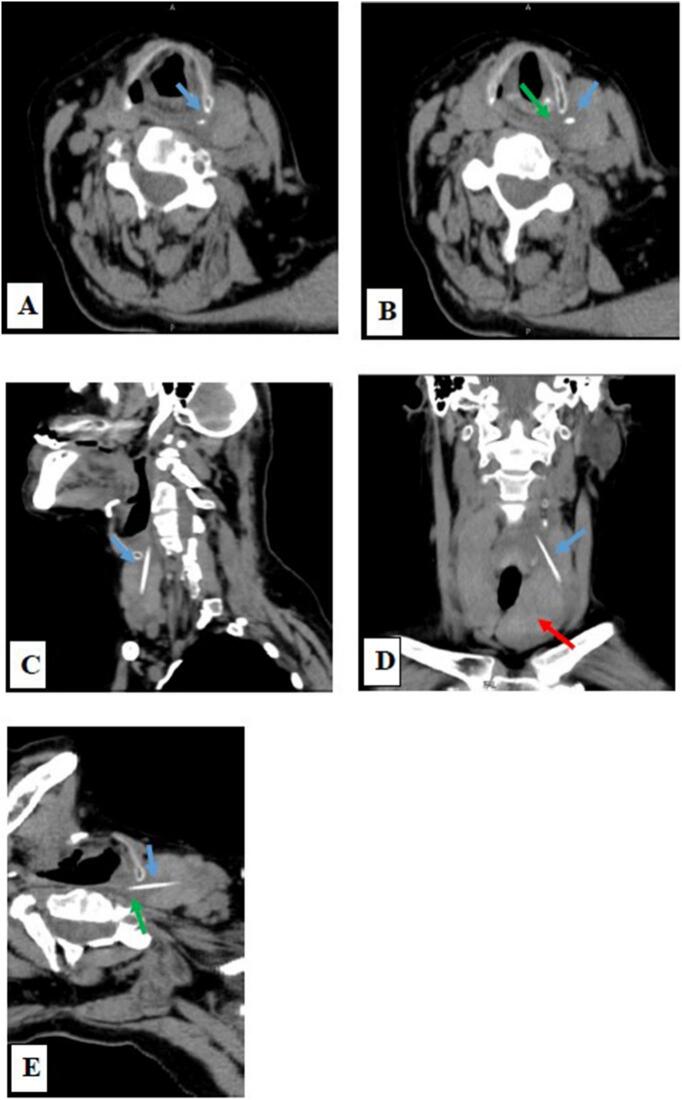
Multiplanar CT images for the neck without IV contrast in the axial section (A and B) revealed a radiodense foreign body shown in Fig. 2. (blue arrow) lodged in the left lobe of the thyroid gland, its upper end abutting the left lateral wall of the hypopharynx at C5 vertebral body level which showed surrounding hypodensity representing the inflammatory reaction of surrounding soft tissue (green arrow) and lower end in the left lobe of the thyroid, no abscess collection or emphysema was noted. C and D: Sagittal and coronal section showed the linear radiodense foreign body (blow arrow) lodged in the left thyroid lobe; the Thyroid gland is enlarged and shows features of multinodular goiter (red arrow). E: The reformatted axial image showed the full length of the foreign body (blue arrow) with surrounding hypodensity along its course representing the inflammatory reaction of surrounding soft tissue (green arrow). (For interpretation of the references to colour in this figure legend, the reader is referred to the web version of this article.)

**Fig. 3:**
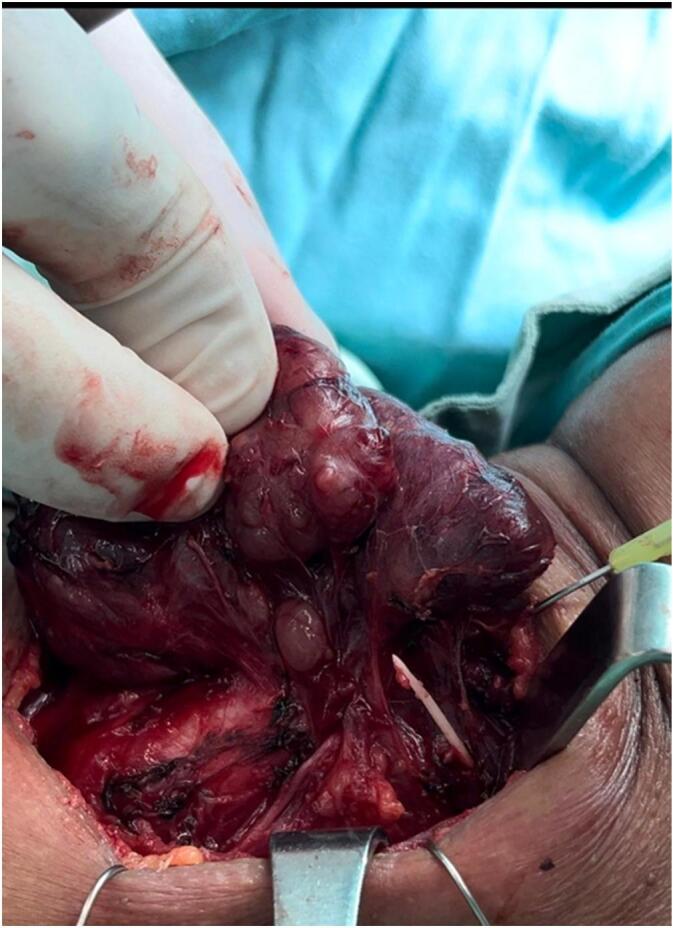
A sharp object is lodged in the postero-medial aspect of the left thyroid lobe after ligation of the left thyroid lobe vessels.

**Fig. 4:**
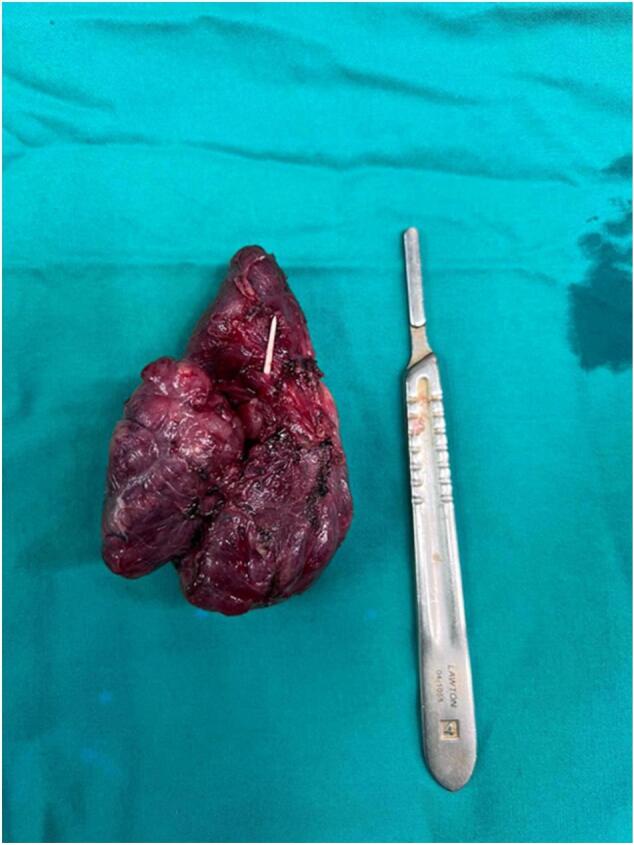
A sharp object penetrating the left thyroid lobe in the posteromedial aspect is shown after left thyroidectomy was performed.

**Fig. 5:**
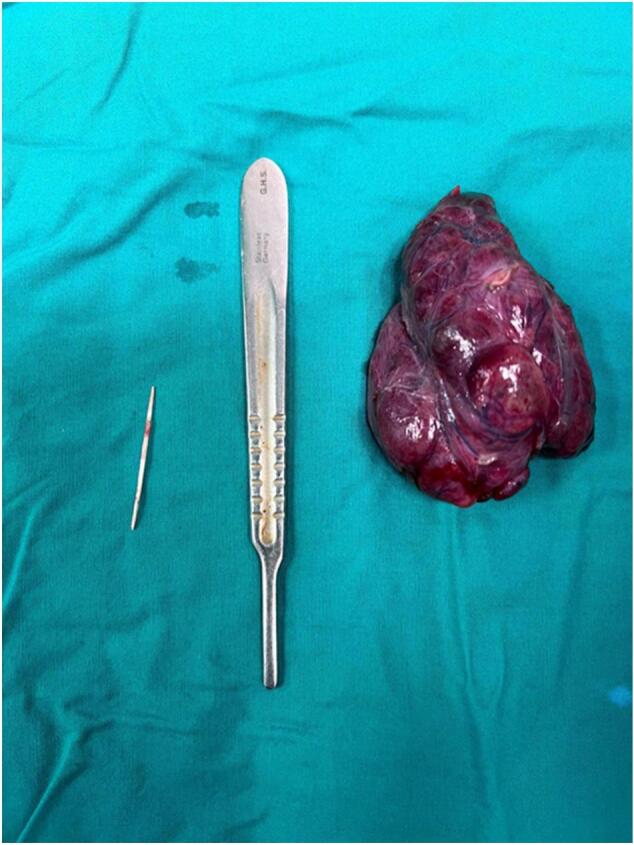
Shows the size of the sharp object.

She underwent neck exploration through a neck collar incision to remove the sharp object. Upon exposing the field; no foreign objects were noted and therefore ligation of the left thyroid lobe vessels was performed to expose the sharp object posteriorly. As expected, it was lodged in the posteromedial aspect of the left thyroid lobe (as shown in Fig. 3), and subsequently, a left hemithyroidectomy was performed as the offending object was located posteriorly behind the upper pole of the left thyroid lobe near the left superior thyroid artery. Careful dissection around pertinent anatomic structures facilitated the identification and removal of the fishbone without complications (Fig. 4, Fig. 5). There were no esophageal, tracheal, or vascular injuries. The patient had an uneventful postoperative course in terms of hypocalcemia, hoarseness, or even wound hematoma. She was discharged home 2 days after the operation. One week later, she followed up in the outpatient clinic with no complaints or further complications.

## 3 Discussions

Hypopharyngeal perforation is uncommon and early recognition is important to avoid its serious complications. The most common causes are extraluminal including penetrating and blunt trauma, or endoluminal from an iatrogenic cause such as diagnostic or therapeutic endoscopy and endotracheal intubation. Foreign body ingestion resulting in hypopharyngeal perforation however is a rare cause [[3], [4], [5]].

The majority of cases of hypopharyngeal perforation due to foreign body ingestion have reported a fishbone as the causative object [4], which is among the most common foreign bodies reported in the upper aerodigestive tract [6].

Signs and symptoms of hypopharyngeal perforation include mostly odynophagia, subcutaneous emphysema, chest or neck pain, hoarseness, stridor, or hemoptysis [7]. Patients may also present with sore throat, dysphagia, and fever as late symptoms of the injury. Fever is often accompanied by retropharyngeal abscess, and subsequently carotid artery pseudoaneurysm formation if left untreated. Serious complications include mediastinitis, pleural empyema, septic shock, and death [8].

The diagnostic approach is unclear for hypopharyngeal perforation in the literature. Clinical assessment along with endoscopy are sometimes enough to make a definitive diagnosis in some cases [9]. Plain radiographs, CT, and contrast swallow scans can be used in combination if there is clinical suspicion of the diagnosis [10]. Neck and chest X-rays are essential initially for the evaluation of foreign bodies or emphysema [11,12]. A CT scan can detect small air collection and is often used to plan an appropriate surgical approach [11,13]. Moreover, Endoscopy helps to detect the presence, site, and extent of the perforation as well as inspects the involved area for pharyngeal edema or hematoma.

The management plan depends on many aspects, including the size and location of the perforation, the patient's hemodynamic status, and the presence or absence of complications [14]. The literature recommends conservative management in patients who are hemodynamically stable with a perforation less than 2 cm in size [14]. Conservative management includes intravenous broad-spectrum antibiotics, parenteral nutrition, and NGT feeding [14] and patients should be followed up to rule out serious complications. Surgical management is performed when there's systemic toxicity, perforations greater than 2 cm, extension to the esophagus, and penetrating injuries [14]. Our patient was managed surgically as she had a foreign body penetrating through the left thyroid lobe perforation.

## 4 Conclusion

This extremely rare case of hypopharyngeal perforation by an ingested fish bone penetrating the thyroid gland itself underscores the importance of maintaining a high index of clinical suspicion and pursuing timely diagnostic imaging in patients presenting with odynophagia or cervical symptoms following foreign body ingestion. Prompt surgical intervention was critical to prevent potentially life-threatening complications like abscess, mediastinitis or vascular injury. Increased clinician awareness regarding this unusual manifestation is essential, and documentation of such rare cases may guide future management strategies to optimize patient outcomes.

## 5 Methods

This work has been reported in line with the SCARE criteria [15].

## Consent

Written informed consent was obtained from the patient for publication and any accompanying images. A copy of the written consent is available for review by the Editor-in-Chief of this journal on request.

## Ethical approval

Ethical approval was not applicable for this study, as there is no formal institutional ethical review board or committee overseeing research activities in the region where the work was conducted.

## Funding

Self-funding, asking for a waive.

## Author contribution

Khalil Abuzaina (1): study concept or design, editor, reviewer, corresponding author.

Sulaiman Fakhouri (2): study concept or design.

## Guarantor

Khalil Abuzaina.

## Conflict of interest statement

There is no conflict of interest.
